# Comparison of two corticosteroid regimens on brain volumetrics in patients with Duchenne muscular dystrophy

**DOI:** 10.1002/acn3.51922

**Published:** 2023-10-12

**Authors:** Sam Geuens, Jeroen Van Dessel, Rosanne Govaarts, Nadine A. Ikelaar, Onno C. Meijer, Hermien E. Kan, Erik H. Niks, Nathalie Goemans, Jurgen Lemiere, Nathalie Doorenweerd, Liesbeth De Waele

**Affiliations:** ^1^ Child Neurology University Hospitals Leuven Leuven Belgium; ^2^ Department of Development and Regeneration KU Leuven Leuven Belgium; ^3^ Department of Neurosciences, Center for Developmental Psychiatry UPC‐KU Leuven Leuven Belgium; ^4^ C.J. Gorter MRI Center, Radiology Leiden University Medical Center Leiden Netherlands; ^5^ Duchenne Center Netherlands Leiden Netherlands; ^6^ Department of Neurology Leiden University Medical Center Leiden Netherlands; ^7^ Department of Medicine Leiden University Medical Center Leiden Netherlands; ^8^ Pediatric Hemato‐Oncology University Hospitals Leuven Leuven Belgium; ^9^ Department Oncology, Pediatric Oncology KU Leuven Leuven Belgium

## Abstract

**Objective:**

Duchenne muscular dystrophy (DMD) is a neuromuscular disorder in which many patients also have neurobehavioral problems. Corticosteroids, the primary pharmacological treatment for DMD, have been shown to affect brain morphology in other conditions, but data in DMD are lacking. This study aimed to investigate the impact of two corticosteroid regimens on brain volumetrics in DMD using magnetic resonance imaging (MRI).

**Methods:**

In a cross‐sectional, two‐center study, T1‐weighted MRI scans were obtained from three age‐matched groups (9–18 years): DMD patients treated daily with deflazacort (DMDd, *n* = 20, scan site: Leuven), DMD patients treated intermittently with prednisone (DMDi, *n* = 20, scan site: Leiden), and healthy controls (*n* = 40, both scan sites). FSL was used to perform voxel‐based morphometry analyses and to calculate intracranial, total brain, gray matter, white matter, and cerebrospinal fluid volumes. A MANCOVA was employed to compare global volumetrics between groups, with site as covariate.

**Results:**

Both patient groups displayed regional differences in gray matter volumes compared to the control group. The DMDd group showed a wider extent of brain regions affected and a greater difference overall. This was substantiated by the global volume quantification: the DMDd group, but not the DMDi group, showed significant differences in gray matter, white matter, and cerebrospinal fluid volumes compared to the control group, after correction for intracranial volume.

**Interpretation:**

Volumetric differences in the brain are considered part of the DMD phenotype. This study suggests an additional impact of corticosteroid treatment showing a contrast between pronounced alterations seen in patients receiving daily corticosteroid treatment and more subtle differences in those treated intermittently.

## Introduction

Duchenne muscular dystrophy (DMD) is characterized by progressive muscle damage resulting from a mutation in the dystrophin gene that disrupts dystrophin function.^1^ Chronic corticosteroid treatment is the primary pharmaceutical option.^2^, ^3^ Prednisone and deflazacort are the two corticosteroids used to treat DMD.^4^ Corticosteroid treatment is considered the standard of care; however, both daily and intermittent dosing regimens exist and are generally chosen based on perceived risk–benefit assessment.^5^

Dystrophin is not only expressed in the muscle, but also in the brain.^6^ In addition to physical symptoms, DMD patients may have a specific behavioral and neurocognitive phenotype.^7^ They are at higher risk for intellectual disability, learning disorders, and psychiatric disorders.^7^, ^8^, ^9^, ^10^, ^11^ Structural brain MRI studies have demonstrated smaller gray matter (GM) volumes and less structured white matter (WM) networks in DMD compared to healthy controls.^12^, ^13^, ^14^, ^15^ Furthermore, these findings provide evidence of the involvement of the brain in DMD, as they suggest that DMD patients with mutations located more distally in the dystrophin gene, and thus lacking brain isoforms, exhibit more pronounced brain alterations. Other imaging modalities, such as arterial spin labeling, resting‐state functional MRI, and PET imaging, have demonstrated differences in cerebral blood flow, spontaneous cerebral activity, and glucose metabolism in DMD patients.^16^, ^17^, ^18^, ^19^ However, the exact relationship between dystrophin expression, brain structure and function, and behavioral and cognitive outcomes remains unclear.^13^, ^20^

Corticosteroids can cross the blood–brain barrier and bind to mineralocorticoid and glucocorticoid receptors to affect gene transcription in all major cell types in the brain.^21^, ^22^, ^23^, ^24^ While studies investigating the cerebral effects of corticosteroid treatment in childhood are scarce, there is evidence that some corticosteroids induce brain atrophy in children and adolescents.^25^, ^26^, ^27^, ^28^ Studies in adults have linked subcortical and cortical atrophy to corticosteroid treatment, as well as decreased WM integrity.^29^, ^30^, ^31^, ^32^, ^33^ Additionally, unfavorable behavioral changes and onset of psychiatric symptoms due to corticosteroid treatment have been described, also in the DMD population.^4^, ^5^, ^34^

As DMD boys are treated with relatively high corticosteroid doses starting from a young age and continuing into early adulthood, they may develop corticosteroid‐induced cerebral alterations on top of DMD‐specific brain pathophysiology. Disentangling both influences is difficult as corticosteroid treatment is the standard of care, and patients not receiving corticosteroids are rare. Alternatively, comparing different corticosteroid regimens may help to understand the differential impact of chronic corticosteroid treatment. Therefore, the main aim of this study was to investigate volumetric brain parameters in DMD patients treated daily with corticosteroids, in DMD patients treated with an intermittent corticosteroid regimen and in healthy age‐matched controls using structural MRI.

## Methodology

### Participants

DMD patients treated daily with deflazacort (cfr. guidelines: 0.90 mg/kg/day with a maximum of 36 mg per day)^2^ were recruited from the neuromuscular reference center at the University Hospitals Leuven (UZL) in Belgium (DMDd, *n* = 20). Inclusion criteria for the DMDd group were 1) age between 9 and 20 years; 2) native Dutch speaking; 3) genetically confirmed diagnosis of DMD with specification of gene mutation; and 4) treatment with daily deflazacort. Participants in the DMDd group were age‐matched with an equal number of DMD patients treated intermittently (10 days on/10 days off) with prednisone (cfr. guidelines: 0.75 mg/kg/day, with a maximum of 30 mg per day)^2^ selected from a historic dataset of 29 patients assessed at the Leiden University Medical Center (LUMC) and described previously^12^ (DMDi, *n* = 20). Finally, a control group consisting of 40 age‐matched healthy participants was included, comprising data from 20 healthy Dutch volunteers from the historic dataset at the LUMC and data from 20 healthy Belgian controls who participated in a similar study published by Van Dessel et al.^35^ at the University Hospitals Leuven.

Ethical approval (UZL: s62667 and LUMC: P09.121) was obtained in both centers. Prior to scanning, participants and parents provided written informed consent. Data management and processing were carried out following the regulations of the Helsinki Convention and the General Data Protection Regulation.

### Demographic and clinical information

Age at time of scanning was collected for all participants, and for DMD subjects additional clinical information was obtained from patient records, including ambulation status, age at corticosteroid initiation, and information on genetic diagnosis. Participants were considered wheelchair bound if they had lost their ability to walk more than 10 meters without support at the time of scanning. Corticosteroid initiation data were available for 20 patients in the DMDd group and 12 patients in the DMDi group, enabling us to calculate the duration of corticosteroid use until the scan date. Information on genetic diagnosis was interpreted to identify participants with mutations upstream of exon 45, which suggests the ability to produce the brain dystrophin isoform Dp140.^8^

### MRI protocol

Structural brain MRI scans were acquired in two centers: UZL scanned the patients in the DMDd group and healthy Belgian controls, while LUMC scanned patients in the DMDi group and healthy Dutch controls. All scans were conducted by trained researchers. Prior to being positioned in the MRI scanner, participants received a comprehensive explanation and were familiarized with the scanner and scan room.

MRI scanning was performed on a 3T Philips Achieva scanner (Philips Healthcare Best, the Netherlands) with a 32‐channel head coil (UZL) or an 8‐channel head coil (LUMC). Structural scans were acquired using a standard T1‐weighted pulse sequence with fixed scan parameters: TE = 4.6 ms, TR = 9.7 ms, flip angle = 8°, field of view = 256 × 242 mm^2^ and 1 mm^3^ voxel size. Scans were assessed by an independent radiologist for gross structural abnormalities.

### Structural analysis

Three voxel wise statistical analyses were performed to assess regional differences in GM between respectively the DMDd group and the control group, the DMDi group and the control group, and the DMDd group and the DMDi group. In each analysis, structural data were analyzed with FSL‐VBM,^44^ an optimized VBM protocol^45^ carried out with FSL tools.^37^ First, structural images were brain‐extracted and GM‐segmented before being registered to the MNI152 standard space using nonlinear registration.^41^ The resulting images were averaged and flipped along the x‐axis to create a left–right symmetric, study‐specific GM template. Second, all native GM images were nonlinearly registered to this study‐specific template and modulated to correct for regional expansion (or contraction) due to the nonlinear component of the spatial transformation. The modulated GM images were then smoothed with an isotropic Gaussian kernel.

**Figure 1:**
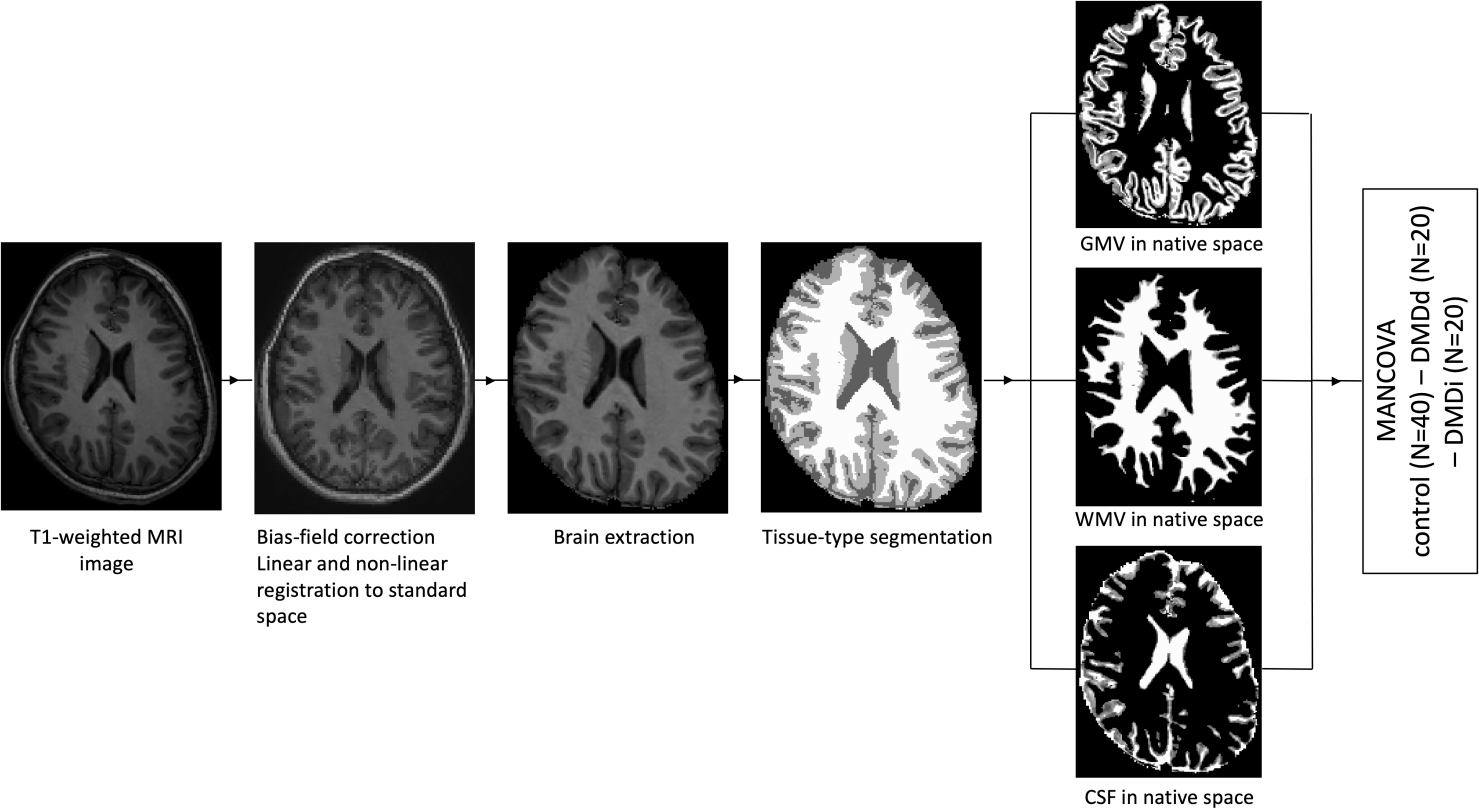
Overview of image processing with the FSL pipeline. Al T1‐weighted images were bias‐field corrected, registered to standard space using a MNI152‐template, and brain extracted before calculating brain volumes in native space.

Figure 1 shows an overview of the consecutive structural preprocessing steps that were performed using the fsl_anat pipeline in FMRIB Software Library (FSL) software v6.0.5^36^, ^37^, ^38^ to calculate the global concentration of intracranial volume (ICV), total brain volume (TBV), GM volume (GMV), WM volume (WMV), and cerebrospinal fluid (CSF). T1‐weighted images were bias‐field corrected, registered to standard space using a Montreal Neurological Institute (MNI) 152‐template (FLIRT^39^, ^40^ and FNIRT^41^), brain extraction (BET^42^ and FNIRT^41^), and tissue‐type segmentation (FAST^43^). Fslstats –V was used to compute each partial volume with the output from FAST.0.1.1. Axial slices were visually inspected to ensure an accurate normalization and segmentation procedure.

### Statistical analysis

Within the FSL‐VBM protocol, voxel‐wise GLM was applied using permutation‐based nonparametric testing, correcting for multiple comparisons across space. Randomise, with 5000 permutations and threshold‐free cluster enhancement (TCFE), was applied with a corrected probability of 0.05 as the significance threshold.

An analysis of variance (ANOVA) was used to test for potential age differences between groups. Next, a one‐model multivariate analysis of covariance (MANCOVA) with ICV, TBV, GMV, WMV, and CSF volume as dependent variables, group as independent variable, and scan site as covariate was performed to test global volumetric differences between groups. As significant differences in ICV were observed, ICV was added as covariate to the MANCOVA in a second analysis to ensure that any observed differences in GMV, WMV and CSF could not be attributed solely to ICV variations. If the result of the MANCOVA indicated a significant difference between the groups, Bonferroni‐corrected post hoc *t*‐tests were performed to explore significant group differences. Statistical analyses were performed in SPSS (version 28, IBM, New York, USA) at a significant level of 0.05.^46^

## Results

### Participants

An overview of the clinical characteristics of the three groups is given in Table 1. No differences were found in age between the three groups. Both DMD groups matched well based on age at start of corticosteroid treatment and duration of corticosteroid treatment.

**Table 1:** Clinical and demographical characteristics.

Characteristic	Control	DMDd	DMDi
No. of participants	40	20	20
Age, year^a^	13.0 ± 2.4	13.2 ± 3.2	13.0 ± 3.1
Age range, year	9.0–18.0	9.5–18.9	9.1–18.3
Scan site, Leuven/Leiden	20/20	20/0	0/20
Wheelchair bound	–	6	13
Age at start of corticosteroid treatment, year^a^	–	6.4 ± 2.1	6.6 ± 2.0
Duration of corticosteroid treatment, months^a^	–	81 ± 30	79 ± 35
Mutation upstream of exon 45	–	6	9

DMD, Duchenne muscular dystrophy; DMDd, treated with daily deflazacort; DMDi, treated with 10 days on/10 days of prednisone.

^a^ Data are presented as mean with standard deviation.

### Brain structure

**Figure 2:**
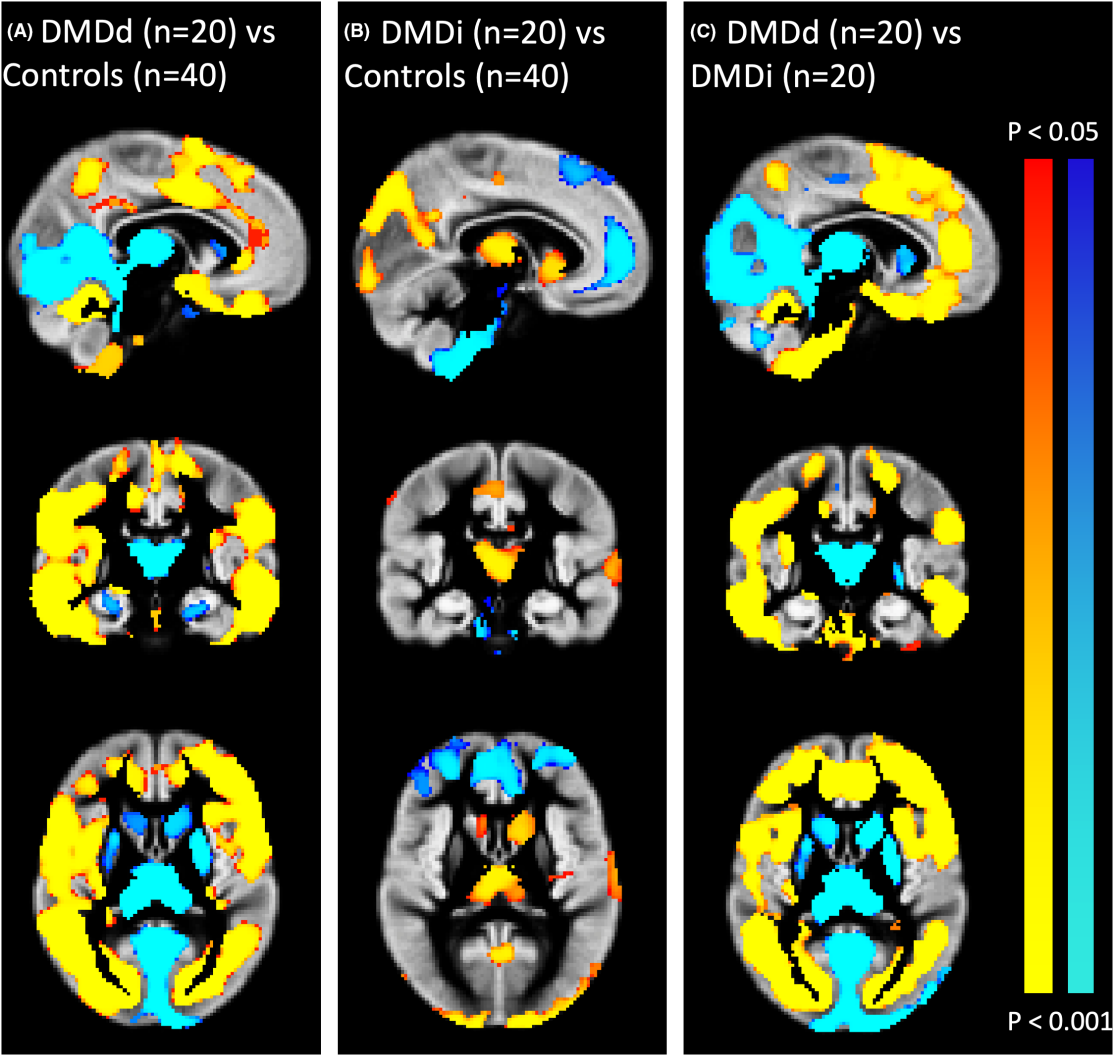
Results of gray matter (GM) voxel‐based morphometry (VBM). (A) Brain regions expressing lower (red‐yellow) or higher (blue) GMV in the DMDd group compared with controls; (B) brain regions expressing lower (red‐yellow) or higher (blue) GMV in the DMDi group compared with controls; (C) brain regions expressing lower (red‐yellow) or higher (blue) GMV in DMDd group compared with the DMDi group. (*p* < 0.05, TFCE‐corrected).

No gross structural abnormalities or incidental findings were discovered with routine visual assessment. VBM analyses revealed that DMDd patients had significantly reduced regional GMV throughout the whole cortex compared to the control group, whereas for the occipital cortex and subcortical structures, the DMDd group showed larger regional GMV. A similar pattern of findings was seen in the comparison between the DMDd group and the DMDi group. Finally, the DMDi group exhibited smaller regional GMV in the occipital cortex and some subcortical structures, as well as higher regional GMV in parts of the prefrontal cortex compared to the control group. Spatial information on volumetric reduction and enlargement is presented in Figure 2.

Further, significant (F(8,146) = 9.750, *p* < 0.001, Wilks' Λ = .425, partial η2 = .348) group differences were observed in global GMV, WMV, ICV, and TBV, and CSF volume (Table 2). The DMDd group showed smaller global ICV (*p* < 0.01), TBV (*p* < 0.001), GMV (*p* < 0.001), and WMV (*p* < 0.01), and higher CSF volume (p = 0.001) compared to the control group.

**Table 2:** Absolute brain volumes of the control, DMDd, and DMDi group.

Characteristic	Control (*N* = 40)	DMDd (*N* = 20)	DMDi (*N* = 20)
Intracranial, cm^3^	1490 ± 104	1358^a^ ^,^ ^b^ ± 71	1479^b^ ± 148
Total brain, cm^3^	1272 ± 87	1094^a^ ^,^ ^b^ ± 69	1259^b^ ± 123
Gray matter, cm^3^	714 ± 47	614^a^ ^,^ ^b^ ± 56	694^b^ ± 56
White matter, cm^3^	558 ± 52	480^a^ ± 34	564 ± 73
CSF, cm^3^	218 ± 31	264^a^ ± 35	220 ± 36

Data are presented as mean with standard deviation.

CSF, cerebrospinal fluid; DMD, Duchenne muscular dystrophy; DMDd, treated with daily deflazacort; DMDi, treated with 10 days on/10 days off prednisone.

^a^ Significant difference with control group after multiple comparison correction.

^b^ Significant difference with other DMD‐group after multiple comparison correction.

**Figure 3:**
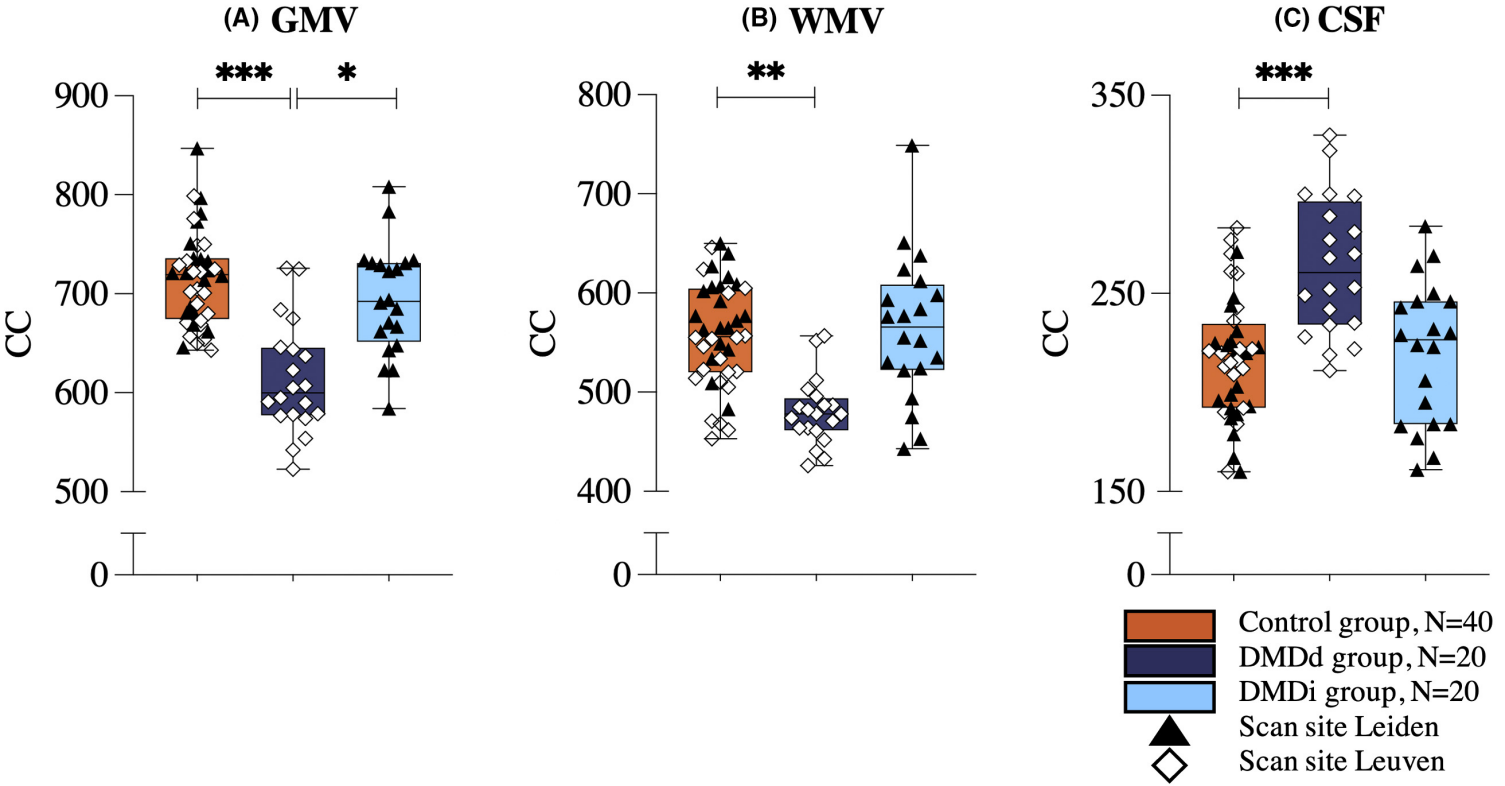
Boxplots of structural brain volumes compared by group. Results of the between‐group post hoc analyses with Bonferroni correction are displayed for gray matter volume (A), white matter volume (B), and cerebrospinal fluid volume (C). Volumes are presented in cm^3^. *p < 0.05; **p < 0.01; ***p ≤ 0.001.

Moreover, the DMDd group had smaller TBV (*p* < 0.05) and GMV (*p* < 0.05) compared to the DMDi group. In contrast, the DMDi group showed no volumetric differences compared with the control group (Fig. 3). Including ICV as a covariate in the analysis did not affect the results. Analyses with only the DMDi (*n* = 12) participants of whom corticosteroid history was available in the medical records did not alter the conclusions.

## Discussion

The present study aimed to examine the effects of different corticosteroid regimens on brain volumetrics in individuals with DMD, revealing more pronounced alterations in brain morphology in patients receiving daily corticosteroid treatment compared to those treated intermittently. This is demonstrated by voxel‐based morphometry analyses which showed a diffuse pattern of regional GM alterations in DMD patients receiving daily corticosteroid treatment, while those treated with an intermittent regimen exhibited regional variations in GMV within specific brain regions. Notably, only the DMD patients receiving daily treatment showed significant volumetric differences in global GMV, WMV, and CSF volume compared to the other groups.

To the best of our knowledge, this is the first study to examine the effect of different corticosteroid regimens on brain volumetrics in patients with DMD. However, numerous studies in other populations have demonstrated an association between exposure to high corticosteroid levels, either endogenous due to Cushing's disease or exogenous due to systemic corticosteroid treatment, and global cerebral atrophy, cortical thinning, and volumetric changes in specific brain regions.^29^, ^30^, ^47^, ^48^ While most of this research has been conducted in adults, a few studies have investigated the impact of corticosteroid treatment on the developing brain in children and found reduced cortical GMV in children treated with daily corticosteroids for epilepsy, rheumatic or nephrotic diseases.^25^, ^26^, ^27^, ^28^ However, it is important to note that the treatment duration for these conditions typically ranges from weeks to months, whereas individuals with DMD receive corticosteroid treatment starting from an early age (4–5 years) continuing into adulthood, resulting in a much longer treatment duration. In addition to GMV loss, recent research has suggested that corticosteroid exposure may impact WM tissue as it affects the proliferation of oligodendrocytes and the structure of myelin.^49^ This may be particularly relevant given that we observed reductions in both GMV and WMV in patients who received daily corticosteroid treatment.

As they have similar pharmacokinetic properties, both deflazacort and prednisone cross the blood–brain barrier and bind to mineralocorticoid (MR) and glucocorticoid (GR) receptors in the brain.^50^, ^51^ Due to chronic corticosteroid exposure, patients treated daily will have a continuous overaction of GR‐mediated processes, whereas MR‐mediated processes may be relatively underactivated, given that synthetic corticosteroids have a relatively lower potency at the MR than endogenous cortisol.^52^ In addition, daily corticosteroid treatment is expected to chronically suppress cortisol levels, which leads to a chronic inhibition of cortisol‐induced natural hormonal processes. In contrast, patients who receive intermittent treatment likely experience periods of low cortisol levels immediately after stopping exogenous corticosteroids, followed by a few days in which natural cortisol production resumes. These hormonal fluctuations may contribute to the mitigation of side effects, which have been shown to be less severe in intermittently treated DMD patients. In order to test this hypothesis, future studies could monitor natural cortisol levels to assess associations with clinical outcomes. In addition, intermittently treated patients should not experience continuous overactivation of the GR, the receptor that is most ubiquitously expressed in the brain, the preferred target of both prednisone and deflazacort, and the receptor that is linked to detrimental glucocorticoid effects on brain structure and function. Thus, the dose regimens may play a role in the observed group differences because of different corticosteroid‐related cerebral effects and differences in interfering with cortisol‐induced brain maturation processes such as synaptogenesis, pruning, and myelination.^25^

A similar interference with developmental processes is demonstrated by studies comparing the physical outcomes of different corticosteroid regimens in DMD, showing that daily administration leads to slowing of growth and delayed puberty induction.^5^, ^53^ In general, daily corticosteroid treatment is associated with more severe side effects compared to intermittent dosing such as weight gain, osteoporosis, cushingoid features, and behavioral changes.^4^, ^5^, ^53^ However, patients treated daily also appear to experience slower functional decline compared to intermittently treated patients.^5^

Previous studies have demonstrated differences in GMV and WMV between individuals with DMD and healthy controls indicating a DMD‐specific and thus dystrophin‐driven cerebral effect. However, none of these studies have specifically investigated corticosteroid treatment as a potential confounding variable contributing to deviant brain parameters. For instance, Preethish‐Kumar et al.^14^ examined WM integrity in two groups of DMD patients with different mutation sites but did not provide details about the specific corticosteroid dosing regimen (daily or intermittent) or its impact on the observed differences. Doorenweerd et al.^12^ discussed the potential role of corticosteroid treatment on their results but included only small subgroups and therefore did not perform separate analyses. Even though we reanalyzed part of the same dataset of that paper in the current study, slightly different results were obtained. Similar findings were observed in terms of regional GMV differences using VBM, but this was not the case for global GM and WM quantified volumetrics. This may be attributed to several methodological differences. First, the present study included only patients treated intermittently who had an age‐match within the daily treated group, resulting in a smaller sample size (two‐thirds) compared to the historic dataset, thereby potentially reducing the statistical power to detect group differences. Second, in contrast with the historic study, the current study implemented preprocessing techniques such as bias field correction, intensity inhomogeneity correction, and registration and normalization to a standard template. These preprocessing steps are required to enable comparisons across groups scanned at different locations,^54^ but the interindividual averaging can result in the loss of fine anatomical details, leading to different results on volumetric outcomes.^55^ Despite the methodological discrepancies between the current study and the historic study, the present findings were consistent in demonstrating subtle regional GMV alterations.

Our study contributes to recent research that focuses on a better understanding of the brain development and function in individuals with DMD.^13^ Brain involvement has been demonstrated in DMD, but as many patients receive chronic high doses of corticosteroids starting from a young age, disentangling the impact of corticosteroid‐induced cerebral effects from DMD‐specific brain comorbidities is difficult. Our findings indicate that corticosteroid exposure and regimen should be considered in future DMD brain studies. The results emphasize the importance of investigating brain, cognitive and behavioral parameters in future clinical trials testing new corticosteroid treatment strategies.

In light of promising new therapeutic strategies and the recent approval of gene therapy for DMD, the role of corticosteroids in this population may evolve. However, it is likely that corticosteroids will continue to be a part of the treatment approach for DMD patients, especially for those who are not eligible for certain therapies based on their genetic mutations. Therefore, ongoing research aimed at mitigating the side effects of corticosteroid use remains of significant clinical importance.

Several limitations should be considered when interpreting the findings of our study. First, as already discussed, daily corticosteroid regimens are linked to more profound effects on growth and physical maturation, but due to incomplete clinical data, our study could not investigate how this is reflected in our results. Group differences in developmental stages could be of influence on our outcomes, and therefore it is recommended for future studies to include physical parameters, such as height, bone age, and Tanner stage, together with cumulative corticosteroid doses to investigate how these factors are linked with each other and with brain volumetrics. Prospective, longitudinal studies could be of great value in order to investigate if and in which way corticosteroid treatment interferes with brain maturation in DMD patients. The present study examines two distinct corticosteroid regimens. However, future prospective investigations may expand this research by incorporating other frequently employed regimens to assess the impact of various corticosteroid dosing schedules on brain maturation in individuals with DMD.

Second, patients within a wide age range (9–18 years) were included. Volumetric changes in different brain regions are normal during human development, with GMV decreasing and WMV increasing during adolescence. The individual developmental rate varies greatly between individuals in the general population, which complicates cross‐sectional comparisons in relatively small cohorts.^56^ Finally, our study design and sample size did not allow to investigate relations between brain parameters and other variables such as mutation site, cognitive abilities, and behavioral outcomes.

In conclusion, our findings indicate that brain volumetrics are more profoundly affected in patients treated with a daily corticosteroid regimen than in those treated intermittently. Moreover, our study highlights the importance of considering the potential impact of different corticosteroid regimens on brain volumetrics in patients with DMD. Future research is needed to elucidate the underlying mechanisms driving these observed differences in brain volumetrics, in order to develop more targeted and effective treatments for DMD patients.

## Author Contributions

SG, EHN, HEK, JL, NG, ND, and LDW were involved in the conception and design of the study. SG, JVD, JL, RG, NAI, HEK, and ND were involved in the acquisition and analysis of the data. SG, JVD, JL, RG, OCM, EHN, HEK, NG, ND, and LDW played an important role in the interpretation of the results. SG, JL, ND, and LDW drafted a significant proportion of the manuscript. All authors reviewed the final manuscript.

## Conflict of Interest

No conflict of interest was reported.
